# CD4^+^ T Cells Play a Critical Role in Microbiota-Maintained Anti-HBV Immunity in a Mouse Model

**DOI:** 10.3389/fimmu.2019.00927

**Published:** 2019-04-30

**Authors:** Tingxin Wu, Fenglei Li, Yongyan Chen, Haiming Wei, Zhigang Tian, Cheng Sun, Rui Sun

**Affiliations:** ^1^Hefei National Laboratory for Physical Sciences at Microscale, The CAS Key Laboratory of Innate Immunity and Chronic Disease, Division of Molecular Medicine, School of Life Sciences, University of Science and Technology of China, Hefei, China; ^2^School of Life Science, Institute of Immunology, University of Science and Technology of China, Hefei, China; ^3^Transplantation and Immunology Laboratory, The First Affiliated Hospital of University of Science and Technology of China, Hefei, China

**Keywords:** hepatitis B virus, commensal microbiota, CD4^+^ T cell, anti-viral immunity, germinal center B cell

## Abstract

The ability of the host to clear hepatitis B virus (HBV) is closely correlated to the establishment of commensal microbiota. However, how microbiota affects anti-HBV immunity is still unclear. Using a well-known hydrodynamical HBV transfection mouse model and treatment with antibiotics (Atb), we explored the change in adaptive immunity (CD4^+^ cells, germinal center B cells and anti-HBs Ab). In our setting, normal mice exhibited complete clearance of HBV within 6 weeks post-hydrodynamic injection (HDI) of HBV-containing plasmid, whereas Atb-treated mice lost this capacity, showing high serum level of hepatitis B surface antigen (HBsAg) without hepatitis B surface antibodies (anti-HBs), similar as what happened in *Rag1*^−/−^ mice or *CD4*^−/−^ mice, suggesting that microbiota may influence the function of CD4^+^ T cells. Furthermore, the numbers of splenic and hepatic effector CD4^+^ T cells (CD44^hi^CD62L^−^CD4^+^ T cells) both decreased with impaired function (IFN-γ synthesis), resulting in lower frequency of germinal center B cells and CD4^+^ follicular helper T cells, and impaired anti-HBs production. We further tried to find the bacterial species responsible for maintaining anti-HBV immunity, and found that each antibiotic alone could not significantly influence HBV clearance compared to antibiotic combination, suggesting that global commensal microbial load is critical for promoting HBV clearance. We also confirmed that TLRs (e.g., TLR2, 4, 9) are not major players in immune clearance of HBV using their agonists and knock-out mice. These results suggest that commensal microbiota play an important role in maintaining CD4^+^ T cell immunity against HBV infection.

## Introduction

Hepatitis B virus (HBV) infection can cause hepatitis, liver cirrhosis, and hepatoma formation. Approximately 350 million people are estimated to be chronically infected with HBV worldwide (1, 2). The International Agency for Research on Cancer within the World Health Organization has classified HBV as a “prominent human carcinogen.” Persistent infection with HBV is related to the age at which HBV exposure occurs. Maternal-neonatal transmission of HBV and acquisition of HBV infection in childhood can both contribute to the chronicity of infection. More than 90% of HBV-exposed neonates and ~30% of children who acquire HBV infections between the ages of 1 year and 5 years cannot clear HBV (3, 4), whereas ~95% of adult-acquired HBV infections are cleared spontaneously. This difference has been postulated to be caused by differences in immunity between young and adult humans. Compared with young humans, adults have a more mature immune system that is more likely to resolve HBV infection.

A recent study has revealed that the establishment of the commensal microbiota is required for the age-related immune clearance of HBV (5). Increasing numbers of studies have shown that the commensal microbiota residing in the gastrointestinal tract “shape” the immune development and function of the host (6–8). Germ-free mice cannot develop normal gut-associated lymphoid tissues (9, 10), they have fewer intraepithelial lymphocytes, and tend to have compromised cytotoxic activity (11). The plasticity of CD4^+^ T cells makes them more inclined to be regulated by the microbiota (after “sensing” signals from the microbiota) and viral infection. In fact, fewer CD4^+^ T cells in the lamina propria have been observed in germ-free mice (12). Besides CD4^+^ T cells, studies in a transfer model of experimental colitis have shown that interleukin (IL)-10 production by regulatory T (T_reg_) cells from germ-free animals is decreased, and the impaired function of T_reg_ cells hinders their ability to prevent disease (13). Furthermore, segmented filamentous bacteria have a crucial role in the coordinated maturation of T helper (T_helper_) cell responses (14). As a “bridge” connecting humoral and cellular immunity, CD4^+^ T cells are crucial for controlling HBV. Virus-specific CD4^+^ T-cell responses may contribute to the association of human leukocyte antigen (HLA)-DR13 with HBV clearance (15). The number of antigen-specific CD4^+^ T cells is significantly increased in patients with acute self-limiting HBV infection compared to patients with chronic HBV infection (16). However, whether CD4^+^ T cells are involved in accelerating commensal microbiota-mediated HBV clearance remains unclear.

We utilized HBV-transfection model by hydrodynamic injection (HDI) of pAAV-HBV1.2 plasmid into adult C57BL/6 (“B6”) mice with depletion of commensal microbiota using antibiotic (Atb)-drinking water. Our results demonstrated that in adult B6 mice, the ability to clear HBV was impaired significantly upon Atb treatment. This impairment was due to a reduction in the number of splenic and hepatic effector CD4^+^ T cells as well as functional impairment of CD4^+^ T cells. In addition, the absence of CD4^+^ T cells abolished the process by which commensal microbiota facilitate HBV clearance. We found that the humoral immune response is impaired after depletion of the commensal microbiota, which was accompanied by inhibition of germinal center (GC) formation and impaired production of anti-HBS. This led to reduced efficiency of HBV clearance. Our results suggest that commensal microbiota play an important role in maintaining efficient CD4^+^ T-cell responses against HBV in the hydrodynamic HBV-transfected mouse model.

## Materials and Methods

Animal protocols were approved by the Ethics Committee for Animal Care and Use of the University of Science and Technology of China (Hefei, China).

### Mice

Male B6 (5–6 weeks old) mice were purchased from the Shanghai Laboratory Animal Center (Shanghai, China). Age-matched *Rag1*^−/−^ mice were obtained from the Model Animal Research Center (Nanjing, China). Age-matched *CD8*^−/−^ and *CD4*^−/−^ mice were kindly provided by Dr. Zhexiong Lian (South China University of Technology, Guangzhou, China). Toll-like receptor (TLR) knockout mice (*TLR2*^−/−^, *TLR4*^−/−^, and *TLR9*^−/−^ mice) were gifts from Dr. Shaobo Su (Sun Yat-sen University, Guangdong, China). All mice were housed in specific-pathogen-free conditions in the Animal Center of the University of Science and Technology of China.

### Plasmids

Both pAAV-HBV1.2 plasmid comprising full-length HBV DNA and pAAV (control) plasmid were kindly provided by Dr. Pei-Jer Chen (National Taiwan University College, Taipei, China). The plasmids used in this study were purified using a NucleoBond^®^ Xtra Midi EF kit (MACHEREY-NAGEL GmbH & Co. KG, Germany) according to the manufacturer's instructions. This kit is used for endotoxin-free purification of plasmid DNA. The endotoxin level in the plasmids was <0.05 EU/μg.

### Mouse Model of HBV Transfection

To establish HBV-transfection model, B6 mice (9–10 weeks) underwent HDI with 6 μg of HBV plasmids via the tail vein within 5–8 s in a volume of phosphate-buffered saline equivalent to 8–10% of the body weight, as described previously (17).

### Treatment

Commensal microbes were depleted using a well-established Atb protocol (18). Four antibiotics, neomycin sulfate (1 g/L), ampicillin (1 g/L), metronidazole (1 g/L), and vancomycin (0.5 g/L), were dissolved in sterile water, which were then supplied as drinking water to the adult mice. B6 mice drank water containing or not containing Atb between 5 and 15 weeks of age. This water was changed twice a week. The body weight of the mice was monitored twice a week. Atb-treated mice that lost >30% body weight were excluded from the study. For the TLRs restoration experiment, Atb-treated B6 mice pre-injected with the HBV plasmid were intra-peritoneally (i.p.) injected with 50 μg Pam3csk4 (InvivoGen, San Diego, CA, USA), 10 μg LPS (Sigma) and 50 μg CpG (Sangon Biotech, Shanghai, China).

### Radioimmunoassays

Serum levels of hepatitis B surface antigen (HBsAg), hepatitis B envelope antigen (HBeAg), and hepatitis B surface antibodies (anti-HBs) were assessed using commercial immunoradiometric assay kits (Beijing North Institute of Biological Technology, Beijing, China) according to the manufacturer's instructions.

### Detection of HBV DNA

Serum HBV DNA copies were detected by quantitative polymerase chain reaction (PCR) using a diagnostic kit for HBV DNA (Amplly, Xiamen, China) according to the manufacturer's instructions.

### Immunohistochemical (IHC) Analyses

Liver tissues embedded in paraffin were cut into 5 μm-thick sections after fixation in 10% neutral-buffered formalin for >24 h. For staining hepatitis B core antigen (HBcAg)^+^ hepatocytes, liver tissue sections were stained by adding rabbit antibodies against HBcAg (Dako, Carpinteria, CA, USA) followed by biotinylated anti-rabbit IgG and streptavidin-horseradish peroxidase conjugates (Zhongshan Goldenbridge, Beijing, China). The stains were developed using a 3, 3′-diaminobenzidine kit (Vector Laboratories, Burlingame, CA, USA).

### Assay to Measure Alanine Aminotransferase (ALT) Activity

ALT activity in serum was determined using an automated Chemray 240 clinical analyzer (Rayto, Shenzhen, China) along with a commercially available kit (Rong Sheng, Shanghai, China) at indicated time points after HDI.

### Cell Isolation

Single-cell suspensions of hepatic mononuclear cells (MNCs) and splenocytes were prepared, as described previously (19). For the isolation of CD4^+^ T cells, splenocytes were separated by magnetic-activated cell sorting using an anti-CD4 monoclonal antibody (Miltenyi Biotec, Bergisch Gladbach, Germany) according to the manufacturer's instructions.

### Flow Cytometry

Several antibodies were used for flow cytometry: fluorescein-isothiocyanate-conjugated anti-CD3 and-GL7; phycoerythrin-conjugated anti-CD8, -CD4, -CD25 and -Fas; phyllochlorin-Cy5.5-conjugated anti-CD44; and brilliant violet (BV)786-conjugated anti-CD19 were purchased from BD Biosciences (Franklin Lakes, NJ, USA). Phycoerythrin-conjugated anti-CXCR5; phyllochlorin-Cy5.5-conjugated anti-IFN-γ, -B220, and -7-AAD; phycoerythrin-Cy7-conjugated anti-NK1.1; allophycocyanin-conjugated anti-CD62L, -CD8 and -PD-1; and allophycocyanin-Cy7-conjugated anti-CD4 were obtained from BioLegend (San Diego, CA, USA). Phyllochlorin-Cy5.5-conjugated anti-Foxp3 was obtained from eBioscience (San Diego, CA, USA). Customized I-A^b^ tetramer loaded with HBsAg_126−138_ peptide (RGLYFPAGGSSSG) was obtained from the NIH Tetramer Core Facility. Freshly isolated MNCs were blocked with rat serum and incubated with fluorescent monoclonal antibodies for 30 min at 4°C. To evaluate HBV-specific CD4^+^T cells, hepatic mononuclear cells or splenocytes were incubated with 10 μg/ml tetramer at 37°C for 1 h in phosphate buffer saline (PBS). The cells were then washed and stained for CD3, NK1.1, CD4, CD8, CD19 and 7-AAD. Tetramer^+^ cells were analyzed in the 7-AAD^−^CD19^−^CD3^+^NK1.1^−^CD4^+^ population. For staining of intracellular IFN-γ, freshly isolated MNCs were stimulated with 50 ng/mL of phorbol myristate acetate (PMA; Sigma–Aldrich, Saint Louis, MO, USA) and 1 μg/mL of ionomycin (Sigma–Aldrich) and treated with 10 μg/mL of monensin (Sigma–Aldrich). After incubation with monoclonal antibodies, the MNCs were fixed, permeabilized, and stained with the intracellular antibody. Data were collected using a flow cytometer (LSR II; BD Biosciences) and analyzed using FlowJo v7.6 (Tree Star, Ashland, OR, USA).

### Quantitative Real-Time PCR

After isolation of splenic CD4^+^ T cells from adult B6 mice treated or not treated with Atb, we measured the mRNA levels of B-cell lymphoma (BCL)-6 and IL-21, as described previously (20). The primer sequences used (forward and reverse, respectively) were: GADPH, 5′-TGG TGA AGG TCG GTG TGA AC-3′ and 5′-CCA TGT AGT TGA GGT CAA TGA AGG-3′; BCL6, 5′-CCT GAG GGA AGG CAA TAT CA-3′ and 5′-CGG CTG TTC AGG AAC TCT TC-3′; IL-21, 5′-ACA AGA TGT AAA GGG GCA CTG T-3′ and 5′-GAA TCA CAG GAA GGG CAT TTA G-3′.

### Statistical Analyses

The unpaired two-tailed Student's *t*-test was used to compare variables between two groups. One-way analysis of variance (ANOVA) was employed to determine significant differences when more than two groups were compared. Mann Whitney *U*-test was used to determine statistical differences in **Figure 4A**. The log rank test was used to determine statistical differences when analyzing the ratio of HBsAg-positive mice in **Figure 4B**. Data are presented as the mean ± standard error of the mean (SEM). *p* < 0.05 was considered significant in all studies.

## Results

### Establishment of Microbiota-Maintained Anti-HBV Mouse Model

**Figure 1:**
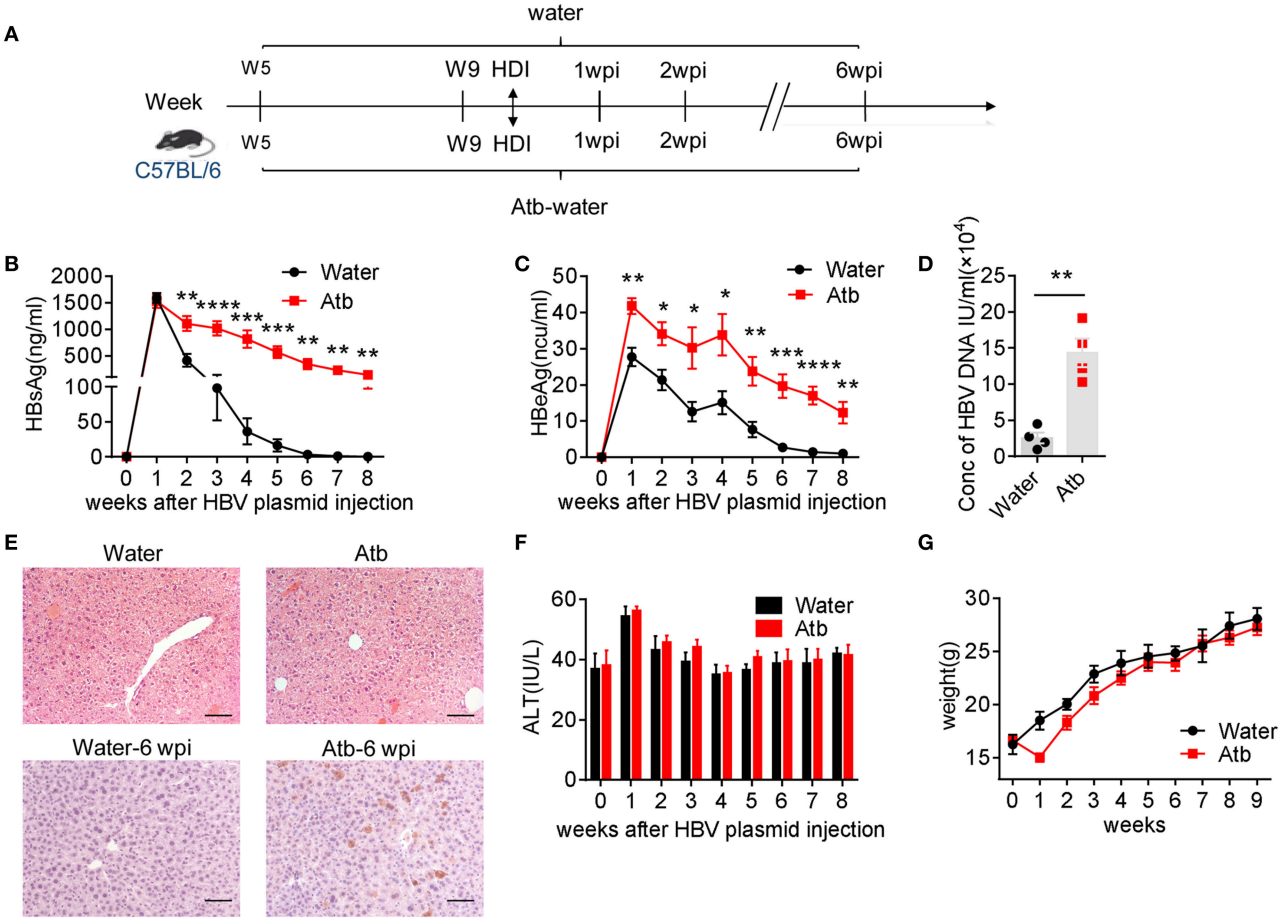
Microbiota depletion delayed HBV clearance. **(A)** Five-week-old B6 mice were divided into two groups: Atb-free and Atb-treated. Mice underwent HDI with 6 μg of HBV plasmids after treatment with Atb-treated water or Atb-free water for 4 weeks. Serum levels of HBsAg **(B)** and HBeAg **(C)** were assessed by immunoradiometric assay at the indicated time points. **(D)** Serum HBV DNA titers were assessed by quantitative real-time PCR at 4 weeks post-injection (wpi) (each point represents one mouse). **(E)** HBcAg^+^ hepatocytes (bottom) in liver tissue were detected by immunohistochemical (IHC) analyses at 6 wpi; H&E staining (top) of samples from Atb-free and Atb-treated mice at time point 0 (at injection) were shown. Scale bar, 50 μm. **(F)** Serum alanine aminotransferase (ALT) levels were quantified using an automated Chemray 240 clinical analyzer at the indicated time points. **(G)** Body weights were recorded at the indicated time points. The data are representative of more than three independent experiments. Results are presented as the mean ± SEM (*n* ≥ 4 mice/group) and unpaired two-tailed Student's *t-*tests were used. ^*^p < 0.05, ^**^p < 0.01, ^***^p < 0.001, ^****^p < 0.0001.

It was reported that low dose of HDI pAAV-HBV1.2 plasmids (<10 μg) via tail vein can create a HBV-carrier mouse model in young C57BL/6 (B6) mice (e.g., <6 weeks old) (17). Interestingly, HBV can be cleared soon if the same treatment is carried out in adult mice (more than 8 weeks old), which is dependent on the existence of gut commensal microbiota (5). However, the immunological mechanisms underlying the gut commensal microbiota-maintained immunity against HBV remains unclear. In the present study, to establish the microbiota-maintained anti-HBV mouse model, we treated mice of 5 and 15 weeks of age with antibiotics (Atb), and then HDI injected 6 μg HBV plasmids at the fifth week after Atb treatment (Figure 1A). As shown in Figures 1B–D, depletion of microbiota impaired the host clearance of HBV, higher serum levels of HBsAg, HBeAg, and HBV DNA were observed in Atb-treated mice. As expected, IHC analyses showed higher expression of HBcAg protein in hepatocytes of Atb-treated mice compared to the control mice (Figure 1E). Of note, Atb treatment alone had no influence on liver inflammation because serum ALT levels were normal at time point 0 (at injection) and HE-staining showed no inflammatory cell infiltration in both Atb-treated and Atb-untreated mice at time point 0 (Figures 1E,F). The serum ALT levels were under normal limits after HDI (Figure 1F). The body weight of Atb-treated mice decreased in the first week but recovered thereafter (Figure 1G). Taken together, these data suggest that Atb-treated adult B6 mice display significantly delayed HBV clearance.

### CD4^+^ T Cells Play a Critical Role in Microbiota-Maintained Anti-HBV Immunity

**Figure 2:**
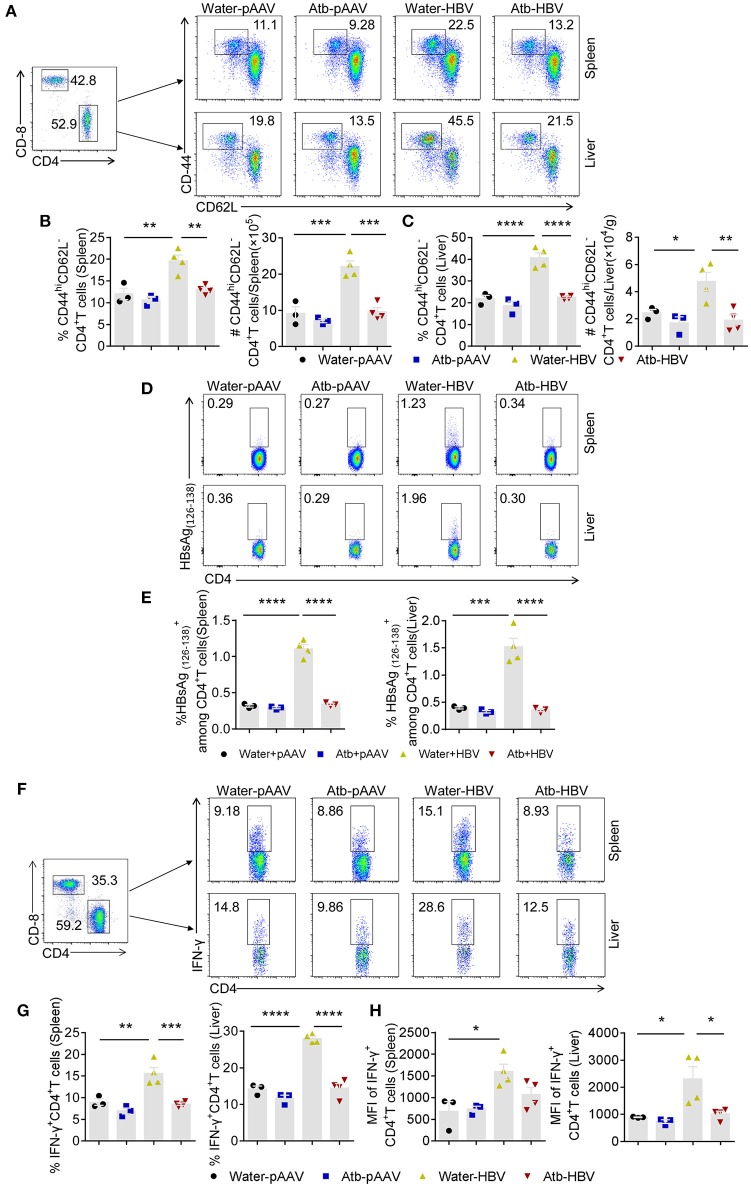
Microbiota depletion reduced amount of effector CD4^+^ T cells. **(A–C)** Mice underwent HDI with 6 μg of HBV plasmids or 6 μg of control pAAV plasmids after receiving Atb in drinking water or Atb-free water for 4 weeks. Splenic and hepatic MNCs were isolated for flow cytometry at 4 weeks post-injection (wpi). **(A)** Dot plots show the percentages of CD44^hi^CD62L^−^CD4^+^ T cells in the liver and spleen. **(B)** Percentage and number of CD44^hi^CD62L^−^CD4^+^ T cells in the spleen. **(C)** Percentage and number of CD44^hi^CD62L^−^CD4^+^ T cells in the liver. **(D,E)** Splenic and hepatic MNCs were isolated for flow cytometry at 6 wpi. **(D)** Dot plots show the percentages of HBsAg ${}_{\left(126-138\right)}^{+}$CD4^+^T cells in the liver and spleen. **(E)** The percentages of HBsAg ${}_{\left(126-138\right)}^{+}$CD4^+^T in the liver and spleen. **(F–H)** Splenic and hepatic MNCs were isolated for flow cytometry at 4 wpi. IFN-γ expression in splenic and hepatic CD4^+^ T cells was detected by intracellular cytokine staining. **(F)** Dot plots show the percentages of splenic and hepatic IFN-γ^+^CD4^+^ T cells. **(G)** Percentages of splenic and hepatic IFN-γ^+^CD4^+^ T cells. **(H)** Mean fluorescence intensity (MFI) of splenic and hepatic IFN-γ^+^CD4^+^ T cells. **(B,C,E,G,H)** Each point represents one mouse. The data are representative of more than three independent experiments. Results are presented as the mean ± SEM (*n* ≥ 3 mice/group) and one-way ANOVA test was used. ^*^p < 0.05, ^**^p < 0.01, ^***^p < 0.001, ^****^p < 0.0001.

Since adaptive immune system plays an important role in HBV clearance, we examined splenic and hepatic MNCs of mice with/without Atb treatment in HDI HBV mouse model by flow cytometry at 4 wpi. No significant differences in the percentage and absolute number of MNCs, CD4^+^ T cells, Treg cells, CD8^+^ T cells, NK cells and NKT cells were observed between the HDI HBV groups and control groups (Supplemental Figures 1, 2). However, the percentage and number of splenic and hepatic CD44^hi^CD62L^−^ effector CD4^+^ T cells after HDI of HBV plasmids were significantly increased compared to controls using pAAV plasmids (Figures 2A–C). Interestingly, the condition in these mice could not be maintained if they were treated with Atb, showing relatively same percentages and numbers of splenic and hepatic CD44^hi^CD62L^−^ effector CD4^+^ T cells as the controls (Figures 2A–C). Flow cytometry revealed ~2-fold lower expression in the spleen (10 vs. 20%; 0.96 × 10^6^ vs. 2.2 × 10^6^) and liver (20 vs. 40%; 1.92 × 10^4^ vs. 4.8 × 10^4^) after Atb treatment (Figures 2A–C). It has been reported that HBV-specific CD4^+^ T cells play an important role in HBV clearance (21, 22). To confirm the presence of HBV-specific CD4^+^ T cells in our model, we isolated splenic and hepatic MNCs to detect HBV-specific CD4^+^ T cells through flow cytometry. The percentages of splenic and hepatic ${\text{HBsAg}}_{\left(126-138\right)}^{+}$CD4^+^ T cells in HBV plasmid-injected B6 mice were significantly higher than those injected with pAAV control, whereas similar percentages of ${\text{HBsAg}}_{\left(126-138\right)}^{+}$CD4^+^T cells have been observed in HBV plamid-injected and pAAV control-injected Atb-treated mice (Figures 2D–E). In addition, MNCs were stimulated with PMA and ionomycin *in vitro* for 4 h and examined by flow cytometry. As expected, HDI of HBV plasmids significantly promoted activation of splenic and hepatic CD4^+^ T cells, showing increased percentages of splenic and hepatic IFN-γ^+^CD4^+^ T cells (Figures 2F, G). However, Atb treatment impaired T cell response against HBV, showing lower percentages of splenic and hepatic IFN-γ^+^CD4^+^ T cells than their control counterparts (9 vs. 15% in the spleen, 14 vs. 28% in the liver) (Figures 2F,G). Furthermore, the mean fluorescence intensity (MFI) of splenic and hepatic IFN-γ^+^CD4^+^ T cells became lower after Atb treatment in HDI HBV model (Figure 2H). These results suggest that depletion of microbiota impairs function of splenic and hepatic CD4^+^ T cells in this mouse model.

**Figure 3:**
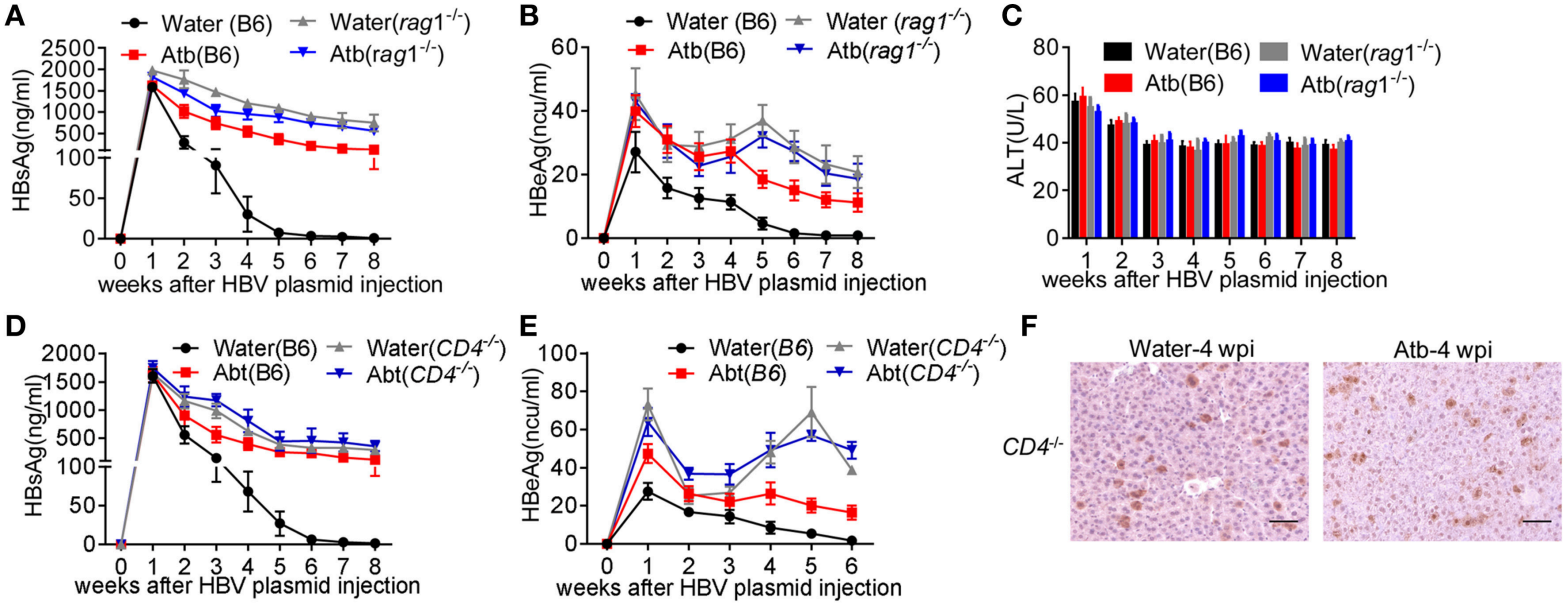
Absence of CD4^+^ T cells impaired HBV clearance. **(A–C)** Five-week-old *Rag*^−/−^ mice were divided into two groups: Atb-free or Atb-treated. Mice underwent HDI with 6 μg of HBV plasmids after receiving Atb-treated or Atb-free water for 4 weeks. Serum levels of HBsAg **(A)** and HBeAg **(B)** were determined by immunoradiometric assay at the indicated time points. **(C)** Serum alanine aminotransferase (ALT) levels were quantified using an automated Chemray 240 clinical analyzer at the indicated time points. **(D,E)** Five-week-old *CD4*^−/−^ *mice* were divided into two groups: Atb-free or Atb-treated. *CD4*^−/−^*mice* underwent HDI with 6 μg of HBV plasmids after receiving Atb in drinking water or Atb-free water for 4 weeks. Serum levels of HBsAg **(D)** and HBeAg **(E)** were determined by immunoradiometric assay at the indicated time points. **(F)** HBcAg^+^ hepatocytes in liver tissue were detected by immunohistochemical (IHC) analyses at 4 weeks post-injection (wpi). Scale bar, 50 μm. The data are representative of more than three independent experiments. Results are presented as the mean ± SEM (*n* ≥ 3 mice/group) and unpaired two-tailed Student's *t-*tests were used.

To confirm the role of adaptive immunity in anti-HBV response, *Rag1*^−/−^mice were injected with 6 μg of HBV plasmids with or without Atb treatment. As shown in Figure 3, Atb-treated B6 mice exhibited impaired anti-HBV immunity by showing higher levels of HBsAg and HBeAg in the serum compared to water-treated B6 mice (Figures 3A,B). More importantly, water-treated *Rag1*^−/−^ mice showed impaired anti-HBV immunity as in Atb-treated B6 mice, and Atb treatment in *Rag1*^−/−^ mice demonstrated no further impairment of anti-HBV immunity (Figures 3A,B). Serum ALT levels were within normal limit and inflammation was absent in controls and Atb-treated mice (both B6 and *Rag1*^−/−^; Figure 3C). These data suggest that contribution of commensal microbiota to HBV clearance is dependent on adaptive immune system.

However, the question that which adaptive immune cell is involved in the microbiota-maintained HBV immunity remains unresolved. Given that significant differences were observed between effector CD4^+^ T cells in Atb-treated B6 mice and water-treated B6 mice (both percentage and number), *CD4*^−/−^ mice were used for further investigation. It was noted that *CD4*^−/−^ mice show higher serum HBsAg and HBeAg levels (Figures 3D,E), comparable to what's been observed in Atb-treated B6 mice (Figures 1B,C; Figures 3D,E) and *Rag1*^−/−^ mice (Figures 3A,B). Importantly, Atb treatment showed no influence on the HBV burden in *CD4*^−/−^ mice (Figures 3D,E). IHC analyses also revealed comparable levels of HBcAg protein expression in hepatocytes of Atb-treated and water-treated *CD4*^−/−^mice (Figure 3F). These results confirm the indispensable role of CD4^+^ T cells in HBV clearance.

### Impaired CD4^+^ T Cell-Supported Anti-HBV Antibody Production by Atb Treatment

**Figure 4:**
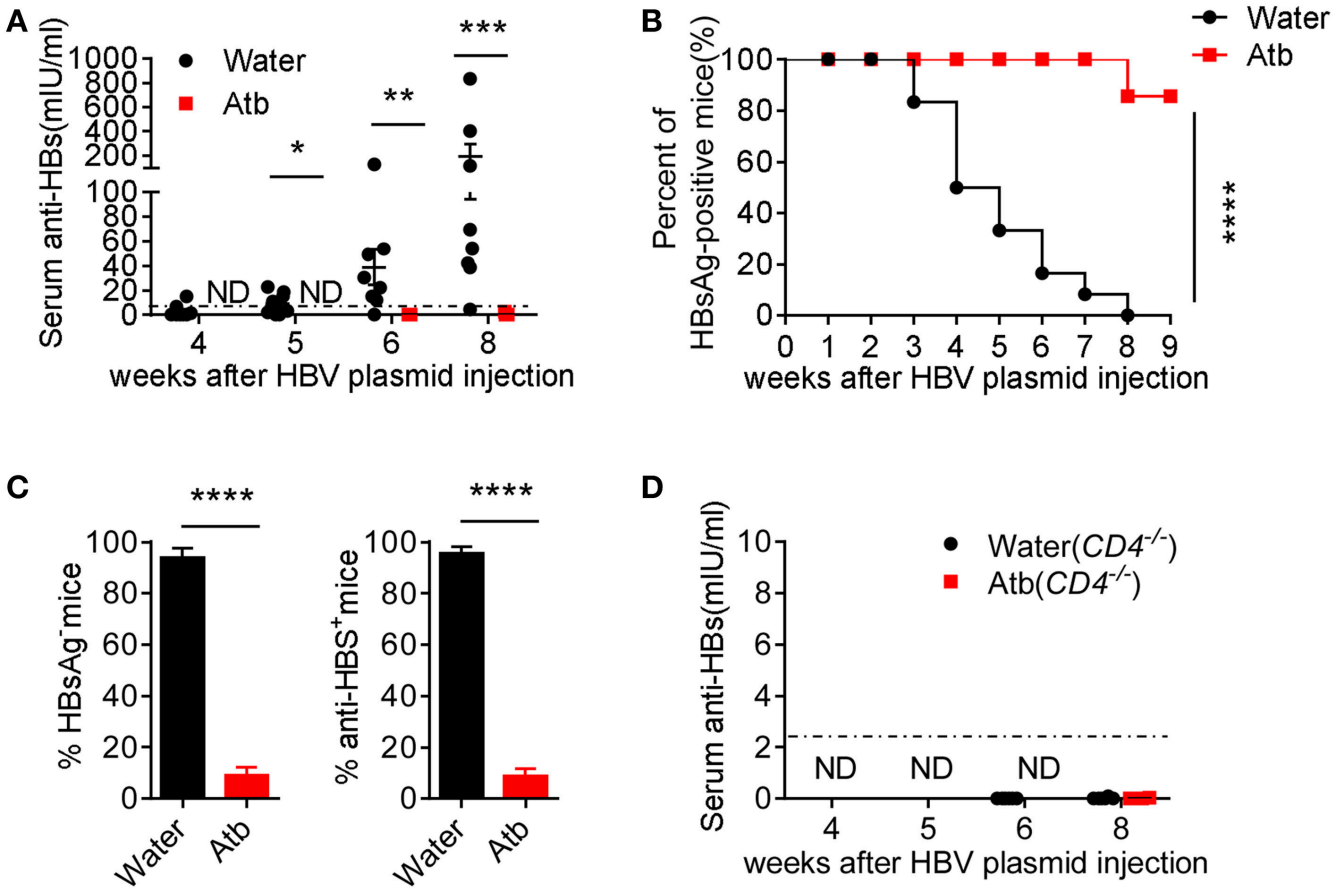
Microbiota depletion reduced antibody production. **(A)** B6 mice underwent HDI with 6 μg of HBV plasmids after receiving Atb-treated or Atb-free water for 4 weeks. Serum levels of anti-HBs were measured by immunoradiometric assay at the indicated time points (The level of anti-HBS below 2.5 mIU/ml is considered as negative) and Mann Whitney *U*-test was used. **(B)** The percentages of HBsAg-positive mice (HBsAg >2 ng/mL) at the indicated time points were calculated and the log rank test was used to determine statistical differences of ratio of HBV-positive mice. **(C)** Percentages of HBsAg-negative (left panel) and anti-HBs production (right panel) in two groups and unpaired two-tailed Student's *t-*tests were used. **(D)** Serum anti-HBs levels were measured by immunoradiometric assay at the indicated time points. The data are representative of more than three independent experiments. Results are presented as the mean ± SEM (n ≥ 4 mice/group). ^*^p < 0.05, ^**^p < 0.01, ^***^p < 0.001, ^****^p < 0.0001.

Antibody plays an important role in HBV clearance (23). Whether CD4^+^ T cells accelerate HBV clearance by helping B cells to elicit antibody production in the presence of commensal microbiota is still obscured. Therefore, we monitored serum levels of anti-HBs at different time points. As expected, Atb-treated mice were unable to produce anti-HBs at 8 wpi (Figure 4A). This is consistent with the serum HBsAg levels that remain positive in Atb-treated mice and sharply decreased in water-treated mice (Figure 1B). We further used serum HBsAg level of 2 ng/ml as a cutoff value to identify HBsAg-positive and HBsAg-negative mice as previously reported (24, 25). We found that the percentage of HBsAg-positive mice in Atb-treated mice is significantly higher than that in water-treated mice within 9 wpi (Figure 4B). Furthermore, ~100% control B6 mice were HBsAg negative and produced anti-HBs Ab; however, only around 10% of Atb-treated B6 mice were HBsAg negative and could produce anti-HBs Ab within 8 wpi (Figure 4C). In addition, *CD4*^−/−^ mice could not produce any anti-HBs Ab regardless of Atb treatment (Figure 4D). Taken together, these data suggest that CD4^+^ T cells are the pre-requirement for anti-HBs Ab production.

**Figure 5:**
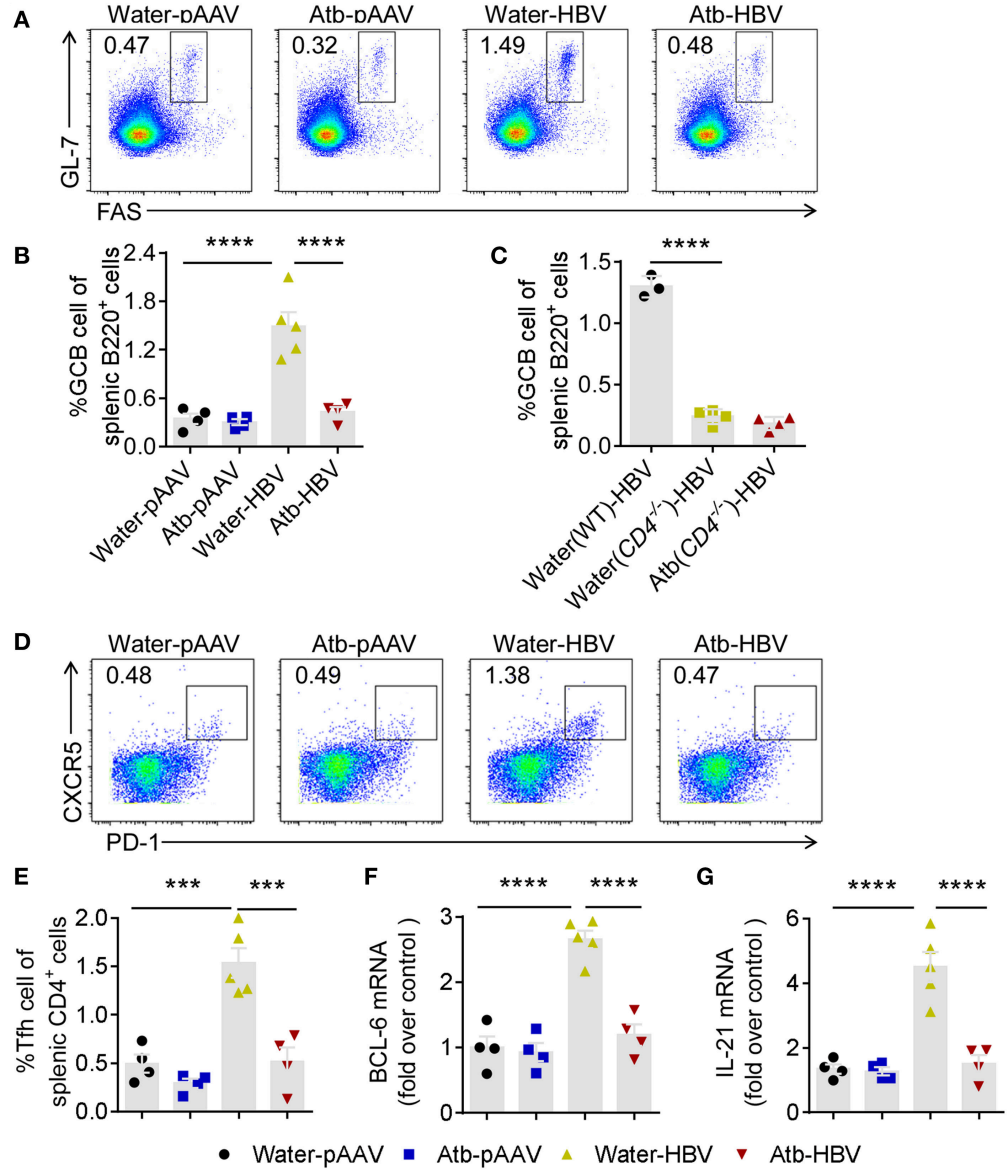
Microbiota depletion impaired B cell response in germinal center. **(A)** Fas^+^GL7^+^ GC B cells (gated on B220^+^ cells) were identified in the spleens of B6 mice 8 weeks after HDI of HBV plasmids or control pAAV plasmids. Representative flow cytometric plots were shown. **(B)** Percentages of Fas^+^GL7^+^ B cells among splenic B220^+^ B cells in B6 mice. **(C)** Percentages of Fas^+^GL7^+^ B cells among splenic B220^+^ B cells in B6 mice and *CD4*^−/−^mice at 6 weeks post-injection (wpi). **(D)** PD-1^+^CXCR5^+^ follicular helper T (T_fh_) cells (gated on CD4^+^ T cells) were identified in the spleens of B6 mice 8 weeks after HDI with HBV plasmids or control pAAV plasmids. Representative flow cytometric plots were shown. **(E)** Percentages of PD-1^+^CXCR5^+^ T_fh_ among splenic CD4^+^ T cells. **(F)** mRNA of Bcl6 in CD4^+^ T cells was analyzed by quantitative real-time PCR. **(G)** mRNA of IL-21 in CD4^+^ T cells was analyzed by quantitative real-time PCR. **(B,C,E–G)** each point represents one mouse. All the data in **(F,G)** were first normalized to GAPDH values and one random sample from group of water-pAAV was set as 1. The data are representative of more than three independent experiments. Results are presented as the mean ± SEM (n ≥ 3 mice/group) and one-way ANOVA test was used. ^***^p < 0.001, ^****^p < 0.0001.

Antibody production is relied on activation of GC B cells. We speculated that HDI of HBV plasmids promoted GC formation and B-cell activation in peripheral immune organs. We therefore detected GC B (Fas^+^GL7^+^ B220^+^) cells in the spleen by flow cytometry at 8 wpi. The percentage of GC B cells in B6 mice HDI with HBV plasmids (≈1.5%) was significantly increased compared to control mice (≈ 0.5%) (Figures 5A,B). In contrast, the percentage of GC B cells in Atb-treated B6 mice HDI with HBV plasmids was similar to that in control mice (≈0.5%) (Figures 5A,B). Furthermore, the percentage of GC B cells in *CD4*^−/−^ mice was significantly lower regardless of Atb treatment (<0.5%) (Figure 5C). Differentiation of B cells in GC is helped by follicular helper T (T_fh_) cells to complete antibody affinity maturation and class switching, we therefore measured the percentage of T_fh_ cells (PD-1^+^CXCR5^+^CD4^+^) in the spleen. The percentage of T_fh_ cells was increased significantly in mice HDI with HBV plasmids (≈1.5%) compared to the control mice (≈0.5%) (Figures 5D,E). However, after Atb treatment, the percentage of T_fh_ cells in mice HDI with HBV plasmids became significantly lower (≈ 0.5%) (Figures 5D,E). Additionally, the mRNA levels of BCL-6 and IL-21, key transcription factor and cytokine of T_fh_ cells, were measured. HBV transfection has caused around 3-fold increase in the levels of BCL-6 and IL-21 mRNA (Figures 5F,G); and around 2-fold decrease in the level of BCL-6 mRNA and around 3-fold decrease in the level of IL-21 mRNA were observed after treatment in mice HDI with HBV plasmids (Figures 5F,G). Collectively, these results suggest that commensal microbiota support the differentiation of GC B cells through CD4^+^ T_fh_ cells, and thereby promote the anti-HBV humoral immunity.

### Global Microbial Load Is Essential for CD4^+^ T Cell-Dependent HBV Clearance

**Figure 6:**
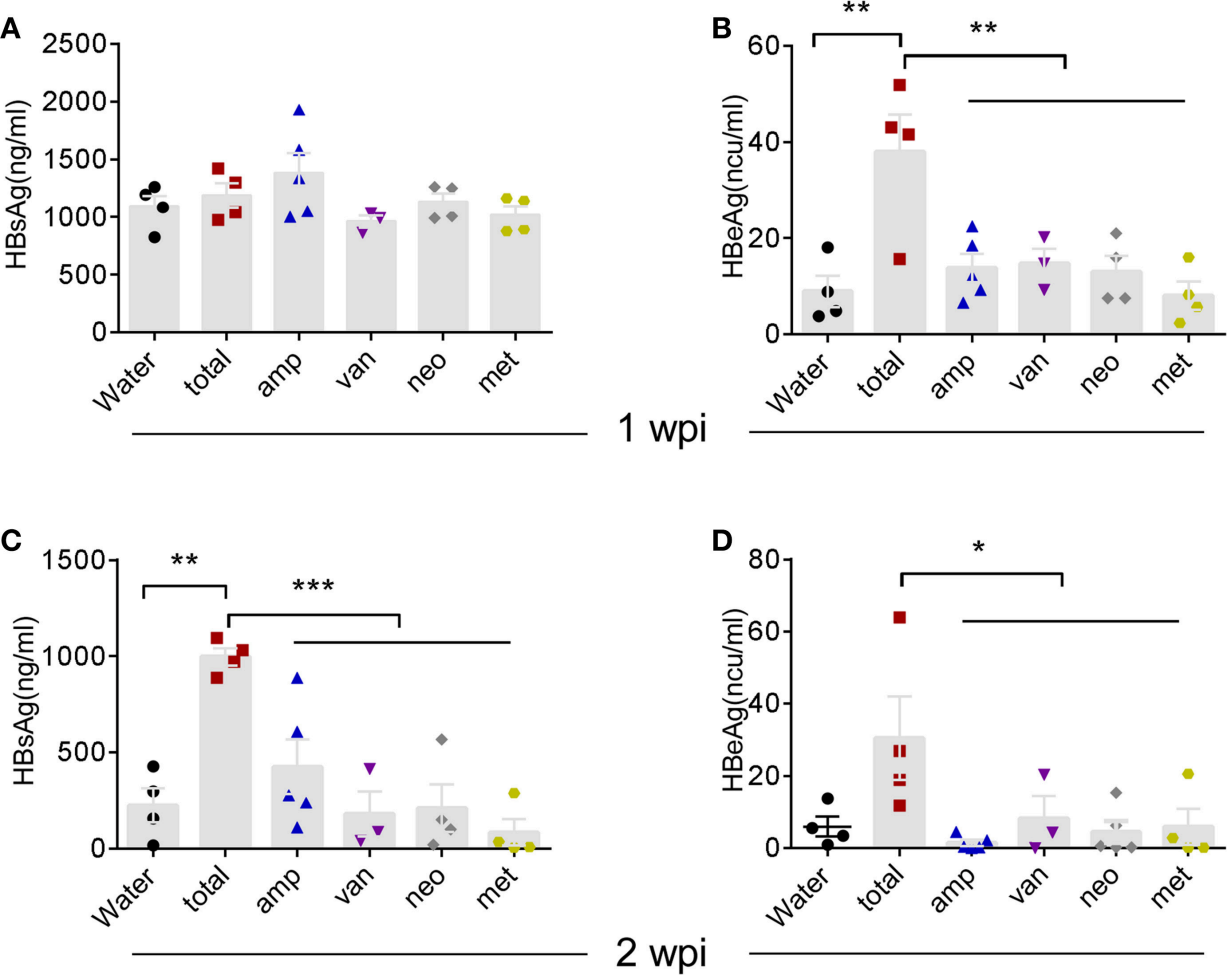
Individual Atb treatment had no influence on HBV clearance. **(A–D)** B6 mice received Atb combination regimen or each Atb alone in their drinking water before HDI of HBV plasmids. Serum levels of HBsAg **(A)** and HBeAg **(B)** were detected by immunoradiometric assay at 1 week post-injection (wpi). Serum levels of HBsAg **(C)** and HBeAg **(D)** were detected at 2 wpi. The data are representative of two independent experiments. Results are presented as the mean ± SEM (*n* ≥ 3 mice/group) and one-way ANOVA was used. ^*^p < 0.05, ^**^p < 0.01, ^***^p < 0.001.

To better understand the bacterial species responsible for stimulating immune responses, we compared the antibiotic combination with each individual antibiotic [neomycin sulfate (neo), ampicillin (amp), metronidazole (met), or vancomycin (van)] in our HDI-HBV model. Interestingly, individual antibiotic showed no significant influence on the HBV clearance compared to antibiotic combination (Figure 6). These data suggest that depletion of the global microbiota impairs HBV clearance possibly through influencing the total load of microbiota without specifically affecting a sensitive species of commensal bacteria.

**Figure 7:**
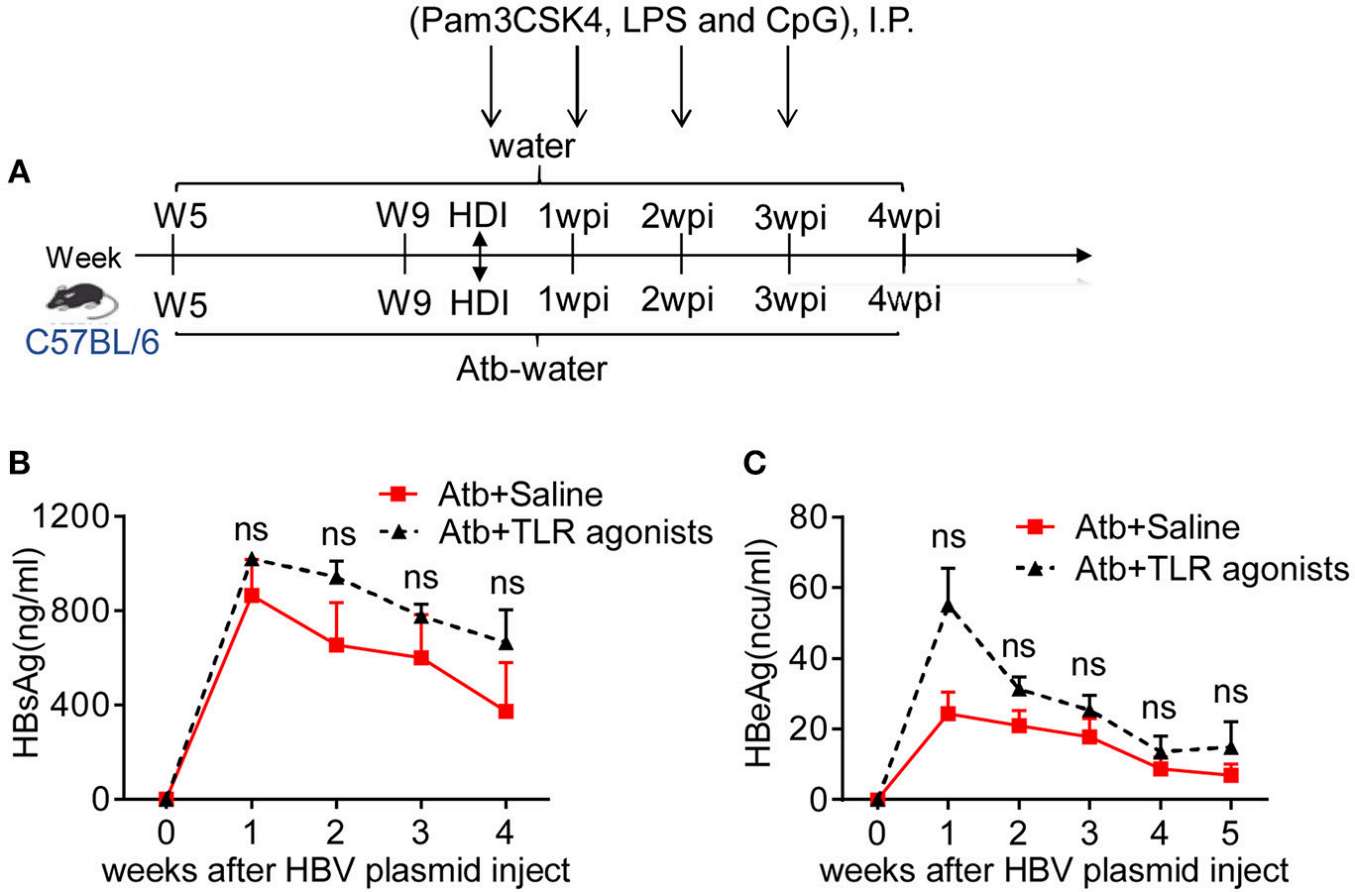
TLR agonists failed to improve clearance of HBV in Atb-treated mice. **(A)** Atb-treated B6 mice pre-injected with HBV plasmids were injected (i.p.) with saline or TLR agonists (Pam3CSK4, LPS and CpG) once a week. Serum levels of HBsAg **(B)** and HBeAg **(C)** were assessed by immunoradiometric assay at the indicated time points (each point represents one mouse). The data are representative of two independent experiments. Results are presented as the mean ± SEM (*n* ≥ 4 mice/group) and unpaired two-tailed Student's *t*-tests were used. ns, not significant.

Commensal microbiota-derived products can provide ligands for TLRs or other pattern-recognition receptors on immune cells and enhance the adaptive immune responses induced by HBV. Emerging evidence has suggested that the TLR2/TLR4/TLR9 pathway is associated with HBV infection (26–29). To ascertain whether such TLR signals are involved in commensal microbiota-accelerated HBV clearance, Atb-treated B6 mice with HDI-HBV were intra-peritoneally injected with TLR agonists Pam3CSK4, LPS, and CpG once a week to activate the TLR2, TLR4, and TLR9 pathways, respectively (Figure 7A). Compared to mice treated with Atb alone, these Atb-treated mice injected with TLR agonists exhibited moderately higher serum levels of HBsAg and HBeAg, however, the differences were not statistically significant (Figures 7B,C). We further utilized adult *TLR2*^−/−^, *TLR4*^−/−^ and *TLR9*^−/−^ mice for constructing HBV transfection models. *TLR2*^−/−^ and *TLR4*^−/−^ mice exhibited similar serum levels of HBsAg and HBeAg as B6 mice and cleared HBV within 4 wpi (Supplemental Figures 3A–D). On the other hand, *TLR9*^−/−^ mice displayed mild delayed HBV clearance compared to B6 mice, prolonged the clearing of HBV from 6 wpi to 8 wpi (Supplemental Figures 3E,F). These data suggest that TLR agonists (e.g., TLR2, 4, 9) are not major players in immune clearance of HBV, and it is necessary to find the major components of microbiota in future study.

## Discussion

Trillions of microbes reside in the guts of vertebrates. The increasing evidence has revealed that the commensal microbiota regulated the immune responses in the gut but are also involved in regulating the immune responses of other organs (e.g., the lungs and liver). In our previous study, young B6 mice displayed HBV persistence in HBV-transfection mouse model. In the present study, adult B6 mice with well-established commensal microbiota displayed rapid clearance of HBV, however, HBV clearance in HBV-transfection mouse model was significantly delayed in Atb-treated adult mice. Our results demonstrate the important role of global microbial load in the spontaneous clearance of HBV and indispensable roles of CD4^+^ T cells and humoral immunity in defense against HBV. Previous studies have shown that the commensal microbiota are associated with the generation, differentiation and function of Treg cells (30–32). But the conclusion is not always consistent. Hall et al. found that the conversion of Treg cells in intestinal tract is inhibited by commensal bacteria-associated DNA (30). Some commensal bacteria have been shown to induce Treg cells and IL-10 production in the gut (31, 32). Our observations suggest that these cells may not be involved in commensal microbiota-dependent immunity against HBV in our model (Supplemental Figures 2A–C). We supposed that the commensal microbiota provided stimulatory signals to activate immune system including Treg cells. The stimulatory signals derived from the commensal microbiota are abolished after depletion by Atb treatment.

The liver has been considered as an important lymphoid organ (33). Its immune cell composition, the special nature of its immune microenvironment, and its regulation of antiviral immune responses are linked to its unique intestinal blood supply containing microbe-derived products and metabolites. The immune response of the liver is closely related to the signals provided by the intestinal commensal microbiota. We showed that the absence of single Atb-sensitive species/group of microflora could not influence the HBV clearance (Figure 6). Since different microbiota compositions were induced by treatment with individual Atb, and such treatment did not result in significant alterations in the commensal bacterial load (34), the unchanged clearance of HBV in individual Atb-treated mice suggests that the global microbial load has a more important role in HBV clearance than the compositions of microbial communities. In addition, it has been reported that the global commensal microbial load (rather than specific Atb-sensitive microflora and the components of microbiota-like TLRs for transduction of the signals derived from the commensal microbiota) is important for maintaining the homeostasis of liver-resident γδT-17 cells (35), a finding that is consistent with our results.

Microbial-derived products include DNA, RNA, endotoxin, exotoxin, etc., making it difficult to find the specific molecules involved in this process. Previous studies have demonstrated that most of these products can be recognized by immune cells through pattern recognition receptors (PRRs) and evoke an immune response. TLRs, as important members of PRRs, have been widely explored. TLRs have been reported to be involved in defense against various pathogens, including HBV (36–39). For example, TLR2 has been reported to be involved in maintaining liver tolerance to HBV (28). Visvanathan et al. found that TLR2 expression was downregulated in HBeAg-positive patients and in HepG2 cells expressing HBeAg (26). Our observation showed that adult *TLR2*^−/−^ mice cleared HBV just as well as adult B6 mice (Supplemental Figures 3C,D), which was in accordance with a recent study showing that TLR2 contributes to HBV persistence (28). Chou et al. reported that age-dependent HBV clearance disappeared with TLR4 mutation (5). Other investigators have suggested that TLR4 signal might activate immune responses against HBV (36, 37, 40). Activation of the CpG-DNA/TLR9 pathway in the “woodchuck” model may contribute to inhibiting HBV (29). TLR9 activation can also enhance hepatic HBV-specific CD8^+^ T-cell responses and lead to HBV clearance in mouse models (41). Therefore, we analyzed the role of TLRs in HBV clearance by using TLR agonists and TLR-deficient mice, we observed that triggering of TLR2, TLR4, TLR9 cannot reverse the delayed clearance of HBV in Atb-treated adult B6 mice (Figures 7B,C). Furthermore, adult *TLR2*^−/−^, *TLR4*^−/−^, and *TLR9*^−/−^ mice kept the capacity to eliminate HBV in a similar manner as adult B6 mice (Supplemental Figures 3A,B). These observations suggest that the impact of TLR2, 4, and 9 on HBV clearance is not significant, indicating that there are other molecules except for TLR2, 4, and 9 might influence the HBV clearance and further studies are needed.

Here, we showed that the commensal microbiota contributed to humoral immunity. This contribution was accomplished by activating CD4^+^ T cells to assist B cells. It has been reported that B cells express receptors that can directly transduce signals derived from the microbiota (42). Microbiota are essential for the B-cell effect, and communication between B cells and microbiota is important for maintenance of intestinal homeostasis (43, 44). Commensal microbiota-driven production of IL-1β and IL-6 induce the generation of regulatory B (B_reg_) cells in the spleen (45). However, the hypothesis that there is a direct interaction between commensal microbiota and B cells, and how B_reg_ cells are involved in the humoral immune responses, need to be investigated further.

In a case-controlled, open-label pilot trial of fecal microbiota transplantation study, HBeAg clearance was observed in patients with chronic HBV infection after long-term antiviral therapy (46). Our results have provided support for this microbiota transplantation in therapeutic intervention for chronic HBV infection. In addition to the role of the commensal microbiota in HBV infection, the role of the commensal microbiota in hepatitis C virus (HCV) infection has also been reported (47, 48). For both chronic HBV and HCV infections, microbiota transplantation combined with routine treatment seems promising. The role of microbiota in contribution to the development and maturation of immune system has been revealed. Our study demonstrates the importance of microbiota in maintaining the function of CD4^+^ T cells against HBV infection. In conclusion, we reveal the critical role of CD4^+^ T cells in commensal microbiota-mediated clearance of HBV using HBV-transfected mouse model, and provide possible cues to use commensal microbiota transplantation in clinical treatment of HBV.

## Ethics Statement

Animal protocols were approved by the Ethics Committee for Animal Care and Use of the University of Science and Technology of China (Authorization number: USTCACUC1601004, Hefei, China).

## Author Contributions

ZT and RS designed the study. TW performed the experiments and analyses. TW, CS, and RS wrote the manuscript. ZT, HW, RS, CS, FL, and YC supervised the study. ZT, RS, and CS critically reviewed the manuscript.

### Conflict of Interest Statement

The authors declare that the research was conducted in the absence of any commercial or financial relationships that could be construed as a potential conflict of interest.
