# Supplementary material for: CD4+ T Cells Play a Critical Role in Microbiota-Maintained Anti-HBV Immunity in a Mouse Model
Source: Front Immunol. 2019 Apr 30;10:927. doi: 10.3389/fimmu.2019.00927 (PMC6503042; doi:10.3389/fimmu.2019.00927)
Supplement: Supplementary file 1 [file Data_Sheet_1.docx]

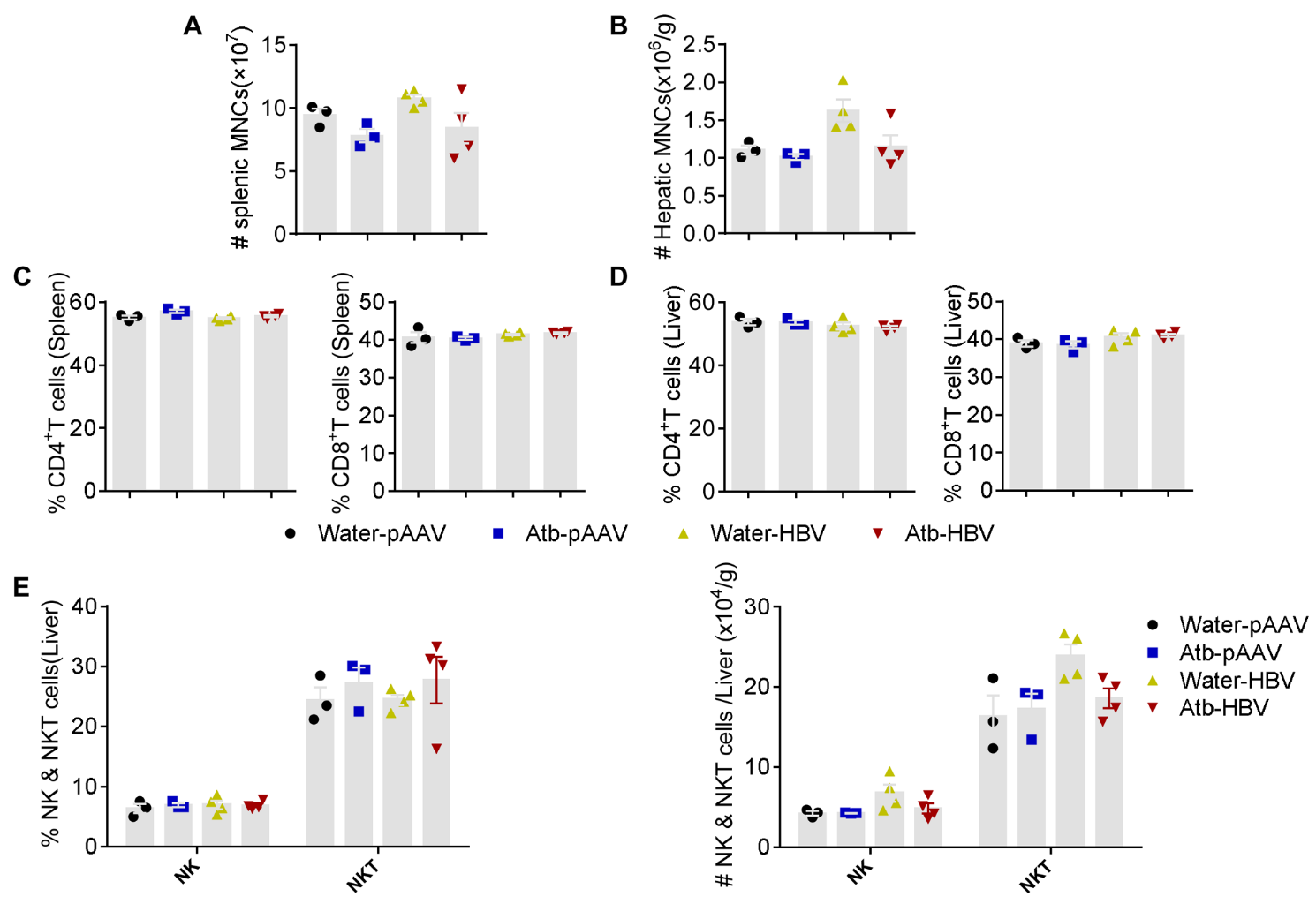


**SUPPLEMENTAL FIGURE 1. Depletion of commensal microbiota had no significant influence on the number of splenic and hepatic MNCs or the percentage of lymphocyte subsets.** (A–D) Mice underwent HDI with 6 μg of HBV plasmids or 6 μg of control pAAV plasmids after receiving Atb in drinking water or Atb-free water for 4 weeks. Splenic and hepatic MNCs were isolated for flow cytometry at 4 weeks post-injection (wpi). (A) Number of splenic MNCs. (B) Number of hepatic MNCs per gram of liver tissue. (C) Percentages of CD4^+^T (CD3^+^CD4^+^) cells and CD8^+^T (CD3^+^CD8^+^) cells in the spleen. (D) Percentages of CD4^+^T cells and CD8^+^T cells in the liver. (E) Percentage (left) or absolute number (right) of NK (CD3^-^NK1.1^+^) cells and NKT (CD3^+^NK1.1^+^) cells in the liver. (A-E) Each point represents one mouse. The data are representative of more than three independent experiments. Results are represented as the mean ± SEM (n ≥ 3 mice/group) and the one-way ANOVA test was used.


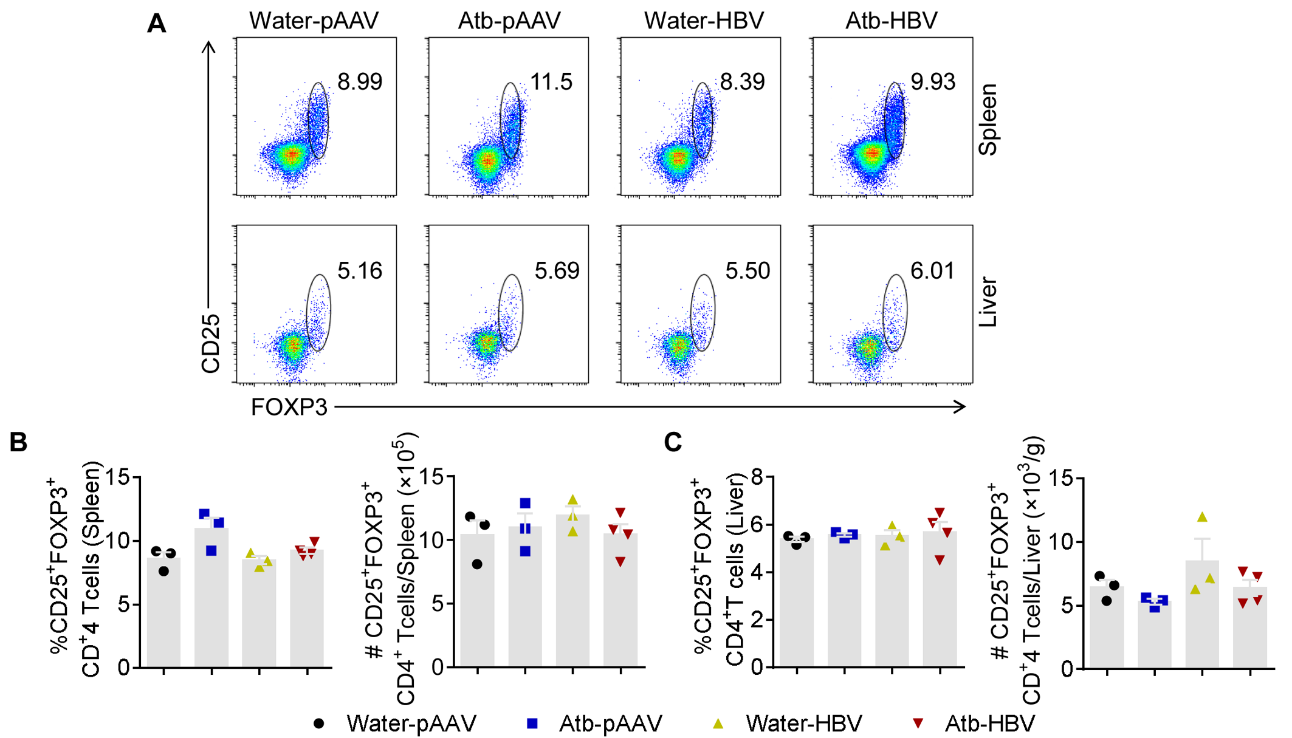


**SUPPLEMENTAL FIGURE 2. Numbers and percentages of splenic and hepatic T_reg_ cells show no significant change after depletion of commensal microbiota**. (A–C) Mice underwent HDI with 6 μg of HBV plasmids or 6 μg of control pAAV plasmids after receiving Atb in drinking water or Atb-free water for 4 weeks. Splenic and hepatic MNCs were isolated for flow cytometry at 4 weeks post-injection (wpi). (A) Dot plots show the percentages of T_reg_ cells (CD3^+^CD4^+^CD25^+^FOXP3^+^) in the spleen and liver. (B) Percentage and number of T_reg_ cells in the spleen. (C) Percentage and number of T_reg_ cells in the liver. (B and C) each point represents one mouse. The data are representative of more than three independent experiments. Results are represented as the mean ± SEM (n ≥ 3 mice/group) and the one-way ANOVA test was used.

**
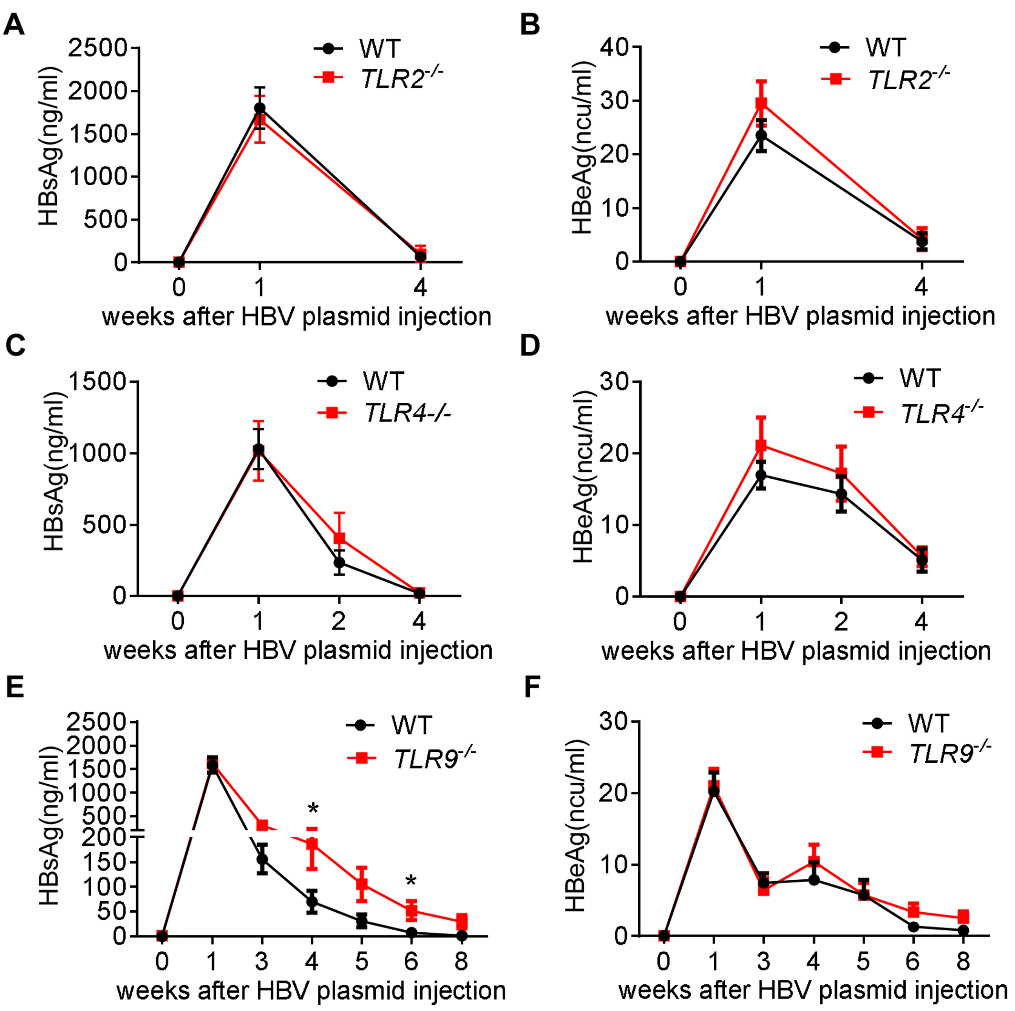
**

**SUPPLEMENTAL FIGURE 3. Deficiency of TLR2/TLR4/TLR9 pathway does not influence the promotion of HBV clearance mediated by the commensal microbiota.** (A–B) Nine-week-old B6 and *TLR2^−/−^* mice underwent HDI with 6 μg of HBV plasmids. Serum levels of HBsAg (A) and HBeAg (B) were determined by immunoradiometric assay at the indicated time points. (C–D) Nine-week-old B6 and *TLR4^−/−^* mice underwent HDI with 6 μg of HBV plasmids. Serum levels of HBsAg (C) and HBeAg (D) were measured by immunoradiometric assay at the indicated time points. (E–F) Nine-week-old B6 and *TLR9^−/−^* mice underwent HDI with 6 μg of HBV plasmids. Serum levels of HBsAg (E) and HBeAg (F) were determined by immunoradiometric assay at the indicated time points. The data are representative of more than three independent experiments. Results are represented as mean ± SEM (n ≥ 5 mice/group) and unpaired two-tailed Student’s *t*-test was used. *p<0.05.
